# Supplementary material for: Magnetic effects of thaumatin crystals; observation of crystal growth by magneto-Archimedes levitation and magnetic orientation
Source: PLoS One. 2025 Feb 13;20(2):e0315335. doi: 10.1371/journal.pone.0315335 (PMC11825019; doi:10.1371/journal.pone.0315335)
Supplement: S1 File — (DOCX) [file pone.0315335.s003.docx]

**Appendix: Crystal Screening**

Crystal screening was carried out in advance to optimize the crystallization condition of thaumatin. All the solutions we used were: (a) thaumatin solution (Maruwa Foods and Biosciences Inc.), (b) 0.4M GdCl_3_ aqueous solution (Wako, Co., Ltd.), and (c) the *precipitant solution* containing 0.2 M *N*-(2-acetamido) iminodiacetic acid-NaOH buffer (pH 6.5), 1.2 M potassium sodium tartrate and 0.04 % NaN_3_ (Maruwa Foods and Biosciences Inc.). As mentioned before, gadolinium makes the solution paramagnetic and is necessary to cause the magneto-Archimedes levitation. The crystallization condition by the batch method was discovered through trial and error of the screening while varying the volume mixing ratio of the solutions (a), (b), and (c). All the screening results are summarized in Tables A and B. Levitated crystals are marked with “Lev”, and non-levitated crystals are marked with “N.L.”.

First, the amount of thaumatin solution was fixed at 45.0 μL, and 0.4M GdCl_3_ aqueous solution was varied to 5.0, 10.0, 15.0, and 20.0 μL, and the precipitant solution was also changed to 31.5, 36.0, 40.5, 45.0, 49.5, 54.0, and 58.5 μL, respectively. As a result, when the 0.4M GdCl_3_ solutions of 15.0 and 20.0 μL were utilized, all the cases failed by the appearance of white precipitation. On the other hand, small crystals appeared when 0.4M GdCl_3_ solutions of 5.0 and 10.0 μL were added. But no crystals were magnetically levitated in such small concentrations of GdCl_3_ solution.

In order to increase the Gd^3+^ ion concentration in relative terms, the thaumatin solution was decreased to 35.0 μL, and the 0.4M GdCl_3_ solution was varied to 10.0, 15.0, 20.0 μL, and the precipitant solution was changed to 31.5, 36.0, 40.5, and 45.0 μL. As a consequence, the magnetic levitation of the crystals was confirmed when the volumetric combination of the 0.4M GdCl_3_ solution and the precipitant solution was 10.0 and 45.0 μL, respectively.

We further decreased the thaumatin volume to 25.0 μL. Then, the 0.4M GdCl_3_ solution was varied to 10.0, 15.0, and 20.0 μL, and the precipitant solution was changed as 31.5, 40.5, 49.5, and 58.5 μL. As a result, we discovered several conditions to realize the magnetic levitation of thaumatin crystals in the cases where the 0.4M GdCl_3_ solution was 10.0 μL and the precipitant solution was 40.5, 49.5, and 58.5 μL.

To confirm the reproducibility hereinabove, a similar screening test was performed by varying the thaumatin volume to 25.0 and 45.0 μL. We varied the 0.4M GdCl_3_ aqueous solution to 10.0 and 15.0 μL, and the precipitant solution was changed to 40.0, 50.0, and 60.0 μL. These results are shown in Table B. In this screening test, the temperature was varied to 12 and 20°C, but the effect of the temperature was small. As a result, large crystals appeared in the condition of 25.0 μL of thaumatin, 10.0 μL of 0.4M GdCl_3_ solution, and 40 μL of the precipitant solution. Based on the above results, we decided to use the combination rate of the solution mixing with 25.0 μL of thaumatin, 10.0 μL of 0.4M GdCl_3_ solution, and 40.5 μL of the precipitant solution throughout our experiment.

**Table A** Screening data of thaumatin crystallization at 20 °C

| thaumatin | 0.4M GdCl_3_ | *Precipitant solution*  containing 0.2 M *N*-(2-acetamido) iminodiacetic acid-NaOH buffer (pH 6.5), 1.2 M potassium sodium tartrate and 0.04 % NaN_3_ | | | | | | |
| --- | --- | --- | --- | --- | --- | --- | --- | --- |
| [μL] | [μL] | 31.5 [μL] | 36.0 | 40.5 | 45.0 | 49.5 | 54.0 | 58.5 |
|  | 5.0 | Un-c | Un-c | Un-c | C-s-m,  N.L. | C-s-m,  N.L. | C-s-m,  N.L. | C-s-m,  N.L. |
| 45.0 | 10.0 | WP | WP | Un-c | C-l (3),  N.L. | C-l (8),  N.L. | C-l (10),  N.L. | C-s-m,  N.L. |
|  | 15.0 | WP | WP | WP | WP | WP | WP | C-s-m,  N.L. |
|  | 20.0 | WP | WP | WP | WP | WP | WP | WP |
|  | 10.0 | C-s-m,  N.L. | C-s-m,  N.L. | C-s-m,  N.L. | C-s-m,  Lev |  |  |  |
| 35.0 | 15.0 | WP | WP | WP | WP |  |  |  |
|  | 20.0 | WP | WP | WP | WP |  |  |  |
|  | 10.0 | WP |  | C-s-m  Lev |  | C-s-m,  Lev |  | C-s-m,  Lev |
| 25.0 | 15.0 | WP |  | WP |  | WP |  | C-l (5),  N.L. |
|  | 20.0 | WP |  | WP |  | WP |  | WP |

WP: white precipitation, C-s-m: small many crystals grown, C-l: large crystals grown, Un-c: solution unchanged, N.L.: not levitated by the magnetic force, Lev: levitated by the magnetic force. Numbers in parentheses indicate the number of large crystals of 0.3 mm or larger.

**Table B** Reproducibility of thaumatin crystallization at temperatures of 20 and 12°C

| temperature | thaumatin | 0.4M GdCl_3_ | *Precipitant solution* | | |
| --- | --- | --- | --- | --- | --- |
| [°C] | [μL] |  | 40.0 [μL] | 50.0 | 60.0 |
| 20 | 45.0 | 10.0 | C-s-m | WP | C-s-m |
|  |  | 15.0 | WP | WP | C-l (11) |
|  | 25.0 | 10.0 | C-l (15) | C-s-m | C-s-m |
|  |  | 15.0 | WP | WP | C-l (6) |
| 12 | 45.0 | 10.0 | C-l (8) | C-l (5) | C-s-m, |
|  |  | 15.0 | WP | WP | C-s-m |
|  | 25.0 | 10.0 | C-l (9) | C-s-m | C-l (7) |
|  |  | 15.0 | WP | WP | C-s-m |

WP: white precipitation, C-s-m: small many crystals grown, C-l: large crystals grown, Un-c: solution unchanged, N.L.: not levitated by the magnetic force, Lev: levitated by the magnetic force. Numbers in parentheses indicate the number of large crystals of 0.3 mm or larger.
