# Supplementary material for: Magnetic effects of thaumatin crystals; observation of crystal growth by magneto-Archimedes levitation and magnetic orientation
Source: PLoS One. 2025 Feb 13;20(2):e0315335. doi: 10.1371/journal.pone.0315335 (PMC11825019; doi:10.1371/journal.pone.0315335)
Supplement: Attachment — Submitted filename: Response to Reviewers25k.pdf [file pone.0315335.s004.pdf]

Response to Reviewers, PONE-D-24-09811

Title

Magnetic effects of thaumatin crystals; observation of crystal growth by magneto-Archimedes levitation and magnetic orientation

The revised parts in response to the comments by the reviewer are highlighted in **red**.

The revised parts by the authors are highlighted in **blue**.

The revised parts where the text is simply moved are highlighted in **green**.

The revised parts according to the Journal editors' requirements are highlighted in **pink.**

Reviewer's comments

[1] Clarification of Motivation: The introduction should better clarify why thaumatin was chosen beyond its industrial sweetness. Emphasize its significance as a model protein in structural biology and its relevance to thermal property measurements.

[Res]

The reasons why we chose thaumatin are that the crystals grow large enough to be visible to the naked eye, its structure is known, and there is little previous research on the effects of magnetic fields. Another major reason is that the transient short hot-wire method can be applied. These were already mentioned in the MS. Only HEWL has been measured for its thermal properties so far. If the thermal properties of another model protein crystal with a known structure are clarified, it will be possible to structurally compare the differences in thermal properties with HEWL and to deeply understand the heat transfer performance from a molecular structural perspective. This will innovate the temperature control system for crystal growth and greatly advance the industrial technology.

In the revised version, the following description has been added to the middle of the introduction.

Page 4, lines 12-15 from the top:

> "If the thermal properties of thaumatin are comparable to those of HEWL, instructive information on heat transfer of other protein crystals can be distinguished by using a molecular structural perspective. This will make a contribution to the progress of industrial technology for controlling crystal growth."

[2] Literature Review: Expand the literature review to include more studies on magnetic effects on protein crystallization, especially those related to thaumatin. This will better contextualize the novelty of your work.

[Res]

We have already cited most of the research papers on the magnetic effects on thaumatin crystallization, but after a detailed search we found one more paper that is related to thaumatin crystallization under

microgravity condition. We newly added that paper in the revised version.

13. Lorber B., Sauter C., Robert M.C., Capelle B., Giegé R., Crystallization within agarose gel in microgravity improves the quality of thaumatin crystals. *Acta Crystallogr D Biol. Crystallogr*. 1999, 55: 1491–1494. DOI: 10.1107/s0907444999008902

[3] Hypothesis and Objectives: Clearly state the hypothesis and main objectives at the end of the introduction. This will guide the reader on what to expect in the subsequent sections.

[Res]

This research is not in the fields of statistics, medicine, or epidemiology, so inductive argumentation, in which a hypothesis is presented and then verified, is not suitable. However, we agree with the point that some comments clarifying our research purpose should be included in the latter half of the introduction.

In the revised version, we added the following sentence about what can be expected if magnetic levitation is realized to thaumatin. This is a comprehensive presentation of the future vision of this research, as clarifying the research objectives.

Page 5, lines 8-10 from the top:

> “If magnetic levitation can be realized in thaumatin crystal growth, we will establish that our method, which has only been successful with HEWL so far, is applicable to the crystallization of other proteins.”

[4] Methodology: Detailed Experimental Protocols: Provide more specific details about the preparation of the buffer solution and the concentration of the precipitant agents used for crystallization. Include information on how the magnetic field gradient was measured and controlled.

[Res]

The procedure for preparing the solution for crystallization is to mix three types of solutions in the optimal concentration ratio. It may seem complicated, but it is actually very simple. The concentration conditions were mentioned in the section of *Crystallization* in page 11.

On the other hand, the concentration of the paramagnetic subject (gadolinium chloride), which works as a driving force on magnetic levitation, is very important. This is necessary to verify whether the levitation occurs or not. Therefore, the concentration after mixing was calculated in the Discussion.

In the revised version, the three types of solutions are labeled as (a), (b), and (c), and the following description has been added. We believe this revision will help readers understand.

Page 11, lines 3-4 from the top:

> “All we had to do was to mix three types of solutions, (a) protein, (b) buffer, and (c) precipitant, in the optimal volume ratio.”

The magnetic flux density and magnetic field gradient $b_z \frac{\partial b_z}{\partial z}$ were referenced in the performance table attached to the magnet. The following description has been added in the revised version.

Page 11, lines 10-12 from the top:

“The magnetic conditions of b_r, b_θ, b_z and $b_z \frac{\partial b_z}{\partial z}$ in the bore were referenced in the performance table attached to the magnet.”

[5] Reproducibility: Include a more detailed description of the experimental conditions to ensure reproducibility. This should cover the exact specifications of the superconducting magnet and any calibration procedures.

[Res]

We agree to the reviewer's request, but it is difficult to ensure reproducibility with biological samples. Protein crystallization is not always reproducible. This is thought to be due to protein degradation or denaturation, but sometimes microcrystals or precipitation occurs. As a countermeasure, we used crystals precipitated in the absence of a magnetic field to confirm in advance whether the crystals would magnetically levitate while observing them with the CCD camera in Fig. 1(c). The magnetic levitation is highly reproducible regardless of the deterioration of protein, because it depends on the magnetic susceptibility and the strength of the magnetic field. Owing to the preliminary experiments in advance, we were able to achieve highly reproducible magnetic levitation on the crystal growth.

In the revised version, the following comments have been added after the final paragraph on page 11 of the manuscript.

“We used crystals precipitated in the absence of a magnetic field to confirm in advance whether the crystals would magnetically levitate, then we observed the magnetic levitation of those crystals with the CCD camera in Fig. 1(c). The levitation is highly reproducible regardless of the deterioration of protein, because it depends simply on the magnetic susceptibility and the magnetic field strength. Owing to such preliminary experiments, we were able to achieve highly reproducible levitating crystal growth.”

In addition, the following sentence has been added to the Appendix on page 21 of the MS.

“Levitated crystals are marked with “Lev”, and non-levitated crystals are marked with “N.L.”.”

[6] Data Presentation: Figures 3 and 4 should be accompanied by more quantitative data, such as measurements of crystal size and growth rates over time. Including graphs or charts would enhance the clarity of the results.

[Res]

Thank you for the advice. We added some sentences presenting the crystal size and growth rates. The revised parts are underlined.

From the 12th line from the top of page 12 to the 6th line from the top of page 13, the section of “*Magnetic levitation of thaumatin crystals*”

“Figure 3(b) shows the growing thaumatin crystals at the meniscus one day later. <u>They were approximately 0.1-0.2 mm in size by appearance</u>. This image presents clear evidence that it is possible to levitate thaumatin crystals by the magneto-Archimedes effect, similar to the mechanism in HEWL

crystals. Figure 3(c) shows the state after four days, revealing that many crystals appeared at the interface as growing. They were approximately 0.3-0.5 mm in size. Figures 3(d), 3(e), and 3(f) are the images after 7, 21 and 48 days, respectively. It was difficult to measure the size of each crystal. The thickness of the crystals further increased to be as thick as to cover up the whole meniscus. Based on these images, the thickness in Figs. 3(d) and 3(e) were about 0.8 and 0.9 mm, respectively, and the maximum thickness in Fig. 3(f) exceeded 1 mm after 48 days. The crystal growth rate was a maximum of 0.2 mm/d up to the first day, and a maximum of 0.1 mm/d from the first to fourth days, but then gradually slowed down, reaching a maximum of about 0.01 mm/d from the 21st to 48th days."

[7] Statistical Analysis: If applicable, provide statistical analysis to support the findings, particularly when comparing the effects of different magnetic field strengths on crystal growth.

[Res]

Statistical analysis is not applicable in this study.

[8] Comparison with Previous Work: Discuss the results in the context of previous studies on HEWL and other proteins. Highlight what new insights your study brings to the field.

[Res]

As for the reviewer's comment of "Comparison with Previous Work", we cannot conduct a comparison of the results because there is almost no analogous research on this research. Regarding the magnetic levitation, the only one we have succeeded in is magnetic levitation growth of lysozyme crystals, which we achieved 20 years ago.

As for the reviewer's comment of "Highlight what new insights your study brings", we would like to respond by slightly amending the conclusion. In the revised version, Conclusion was partially modified as:
Page 19-20.

"We examined the magnetic effects of thaumatin crystals. We succeeded in realizing the magnetic levitation of thaumatin crystals by the technique of magneto-Archimedes effect, and demonstrated the crystal growth in situ under a containerless condition. These results are noteworthy in proposing that the containerless crystallization, which has only been successful with HEWL so far, is also applicable for thaumatin.

We observed the growing process of thaumatin chronologically and recorded the process in a short movie. We also presented a visualization of the magnetic orientation of thaumatin crystals under 6.0 T. In addition to that, we carried out a structural analysis of the thaumatin crystal. Through the refinement of the structure, we could not recognize the clear electron densities as gadolinium ions, supporting the fact that the magneto-Archimedes levitation of thaumatin is free from coordinate bonds to the paramagnetic substance of gadolinium. In other words, the paramagnetic substance of the aqueous precipitant solution may help only to promote the crystals' levitation, having little effect on thaumatin crystallization. Whatever happens, the magnetic levitation and containerless crystallization are highly versatile

technologies and have potential for producing quality protein crystals. The findings on magnetic effects hereinabove also have academic value in contribuitng to the progress of new technical fields of protein crystal growth with pioneering engineering.”

[9] Mechanistic Insights: Provide a more in-depth discussion of the underlying mechanisms of magneto-Archimedes levitation and magnetic orientation. Explain why the effects observed in thaumatin crystals differ from those in HEWL.

[Res]

That is also explained in the section on *Magneto-Archimedes levitation* in Theoretical Ideas. More details on the magneto-Archimedes levitation have also been described in our previous study [21]. The levitation principle observed in thaumatin crystals was almost the same as that in HEWL. The precipitation styles were also very similar to each other. The only difference was the magnitude of paramagnetic susceptibility in the protein solution. As proof of this, the meniscus of the thaumatin solution in Fig. 3 was not horizontal. This is because the paramagnetic susceptibility of thaumatin solutions was low. In the case of HEWL, the solution is strongly attracted downward by the magnetic force, so the meniscus is deformed and becomes almost horizontal in the magnetic field.

The revised version adds the following statement in the section of *Magnetic levitation of thaumatin crystals*.

Page 12, lines 9-12 from the top:

> “In the case of lysozyme levitation, the concentration of paramagnetic substance in the solution was high, so the solution was strongly pulled downward by the magnetic force, causing the meniscus to deform and became almost horizontal in the magnetic field [22].”

In addition, the revised version adds the following description about magnetic orientation in the section of *Magnetic orientation of thaumatin crystals*.

Page 15, lines 9-11 from the top:

> “Since all the crystals were grown oriented from the beginning, the magnetic orientation of thaumatin is found to be possible at 6 T, smaller than 10-11 T.”

Page 15, lines 12-13 from the top:

> “We assume that the levitated crystals must be oriented, although the presence of the orientation can not be observed in the photograph of Fig. 3.”

Journal editors’ requirements

[10] **Funding Information and Financial Disclosure sections**

We note that the grant information you provided in the ‘Funding Information’ and ‘Financial Disclosure’ sections do not match. When you resubmit, please ensure that you provide the correct grant numbers for the awards you received for your study in the ‘Funding Information’ section.

[Res]

Sorry for the confusion. We did not receive any fundings from the US government or international research institutions for this research, but we did receive academic support from the Japanese government (MEXT/JSPS, KAKENHI). We removed the information about the research grant from the attachment, and listed it in the Financial Disclosure in Funding Information.
MEXT/JSPS, KAKENHI, Grant Number JP15K04669.
MEXT/JSPS, KAKENHI, Grant Number JP20K04335.

[11] In the online submission form you indicate that your data is not available for proprietary reasons and have provided a contact point for accessing this data. Please note that your current contact point is a co-author on this manuscript. According to our Data Policy, the contact point must not be an author on the manuscript and must be an institutional contact, ideally not an individual. Please revise your data statement to a non-author institutional point of contact, such as a data access or ethics committee, and send this to us via return email. Please also include contact information for the third party organization, and please include the full citation of where the data can be found.

[Res]

The comment, "All the data in this study are available from the corresponding author upon reasonable request" is often seen in the journals. Now the contact point was changed to the research institute at the university to which the corresponding author (Syou Maki) belongs.

[12] Please include captions for your Supporting Information files at the end of your manuscript, and update any in-text citations to match accordingly.

[Res]

We changed it as the editors said.

Page 22:

"We will make two short movies available to the public. "Short movie of Figure 4" verified the magnetic levitation of thaumatin crystals by changing the magnitude of the magnetic force, where $b_z \frac{\partial b_z}{\partial z}$ was 1100 T^2/m in the vicinity of the upper coil edge. This is recorded in real time speed.

The other, "Short movie of Figure 5", made clear the chronological change of the crystal growth observed from the vessel top in the horizontal bore system, where the magnetic flux (6.0 T) is directed upward in the picture. This movie is edited at 20,000 times speed. Both movies were taken at 20°C. "

In addition, we added a comment about these short movies in the caption of Figure 4 and Figure 5.

Others

[13] Figure 1(b) was modified.

[14] In the Introduction, we added the following sentence to present why we attempted the structural analysis of thaumatin crystals.

Page 5, lines 12-16 from the top:

"As far as we know, there has been no previous research on using a paramagnetic substance to crystallize thaumatin, and it is also unknown whether or not such substances can bond to thaumatin molecules. Our investigation will provide detailed insight into the effect of the paramagnetic substance on magnetic levitation."

[15] In the section of "*Equipment for the magnetic orientation*", the second line from the first paragraph beginning seemed to be verbose, so we modified as:

Page 9, line 1 from the bottom:

"….capillary tube. In this study, we crystallized thaumatin by the so-called "hanging drop method", in ……"

[16] Information on the model of a light source has been added.

Page 10, line 9 from the top:

"a light (LA-100USW, Hayashi-Repic Co. Ltd.)."

[17] The number of water molecules was changed from "335" to "345". This change came about as the result of examining information about the water molecules contained in the asymmetric unit of the most recent thaumatin crystal structure deposited in the PDB.

Page 17, line 3 from the top:

"We confirmed that 345 water molecules and ……"

[18] The beginning comment of the caption of Figure 5 was missing, so we corrected it.

[19] Dr. Masao Fujiwara, one of the coauthors, retired from Hiroshima University at the end of March 2024. His latest email address has been changed to the following:

fuujin.raijin.world@ae.auone-net.jp
