# Supplementary material for: Magnetic effects of thaumatin crystals; observation of crystal growth by magneto-Archimedes levitation and magnetic orientation
Source: PLoS One. 2025 Feb 13;20(2):e0315335. doi: 10.1371/journal.pone.0315335 (PMC11825019; doi:10.1371/journal.pone.0315335)
Supplement: Attachment — Submitted filename: Response to Reviewers49.pdf [file pone.0315335.s005.pdf]

Response to Reviewers, PONE-D-24-09811

Title

Magnetic effects of thaumatin crystals; observation of crystal growth by magneto-Archimedes levitation and magnetic orientation

The revised parts in response to the comments by the Reviewer 3 are highlighted in **red**.

The revised parts by the authors are highlighted in **blue**.

The revised parts pointed out by the Editors are highlighted in **green**.

Revisions by the comments of the Reviewer #3: Comment for authors:

[1] To enhance readability the authors should simplify the complex sentences, and eliminate unnecessary terminology, such as “strictly speaking”.

[Res.]

In the previous version, we used the phrase “strictly speaking” only once, in the line 103 of page 7 for the explanation of the roles of the component f_r. As we mentioned in the text, few magnetic levitation studies have focused on the effect of f_r. This is because the magnitude of f_z is overwhelmingly larger than that of f_r in a conventional solenoidal superconducting magnet. However, we think the effect of f_r is very important practically. We observed that even a small force works as a definite driving force in a pseudo-weightless state. In my previous research on magnetic separation of sucrose and glucose, we could visualize an interesting phenomenon in which those particles fall along an hourglass-like trajectory due to the influence of f_r in the weightless state during the fall [30]. We also achieved the first successful magnetic levitation of crystals by tilting the bore horizontally and using a vertical f_r [31]. Thus, the role of f_r cannot be ignored, and therefore we think there is no need to delete the phrase “strictly speaking” in the text. However, it might be possible that the reviewer felt that phrase too strong, so we changed it to “More particularly”, instead.

In addition, we also added the following sentence in Page 7, Lines 113-116, and newly used some papers of Ref [30, 31] to the reference.

“We confirmed that the effect of f_r could become tangible through the techniques of magnetic separation in the weightless condition [30] as well as the experiment for thermal properties of HEWL crystals [31]”

30. Maki S., Hirota N., Magnetic separation technique on binary mixtures of sorbitol and sucrose, *J. Food Eng.* 2014, 120 C: 31–36, DOI: 10.1016/j.jfoodeng.2013.07.006
31. Maki S., Tanaka S., Miyagi K., Mori T., Isaka Y., Hagiwara M., Fujiwara S., Thermal conductivity and thermal diffusivity of lysozyme crystals, the *c*-axis of which is magnetically orientated along the direction of the probe wire, *Exp. Heat Transf.* 2023, 36 (4), DOI: 10.1080/08916152.2023.2197903

[2] Please reorganize the introduction into paragraphs that flow logically from the background to specific

research questions.

[Res.]

One of the main results of this paper is the successful magnetic levitation of thaumatin. However, the introduction did not explain much about the significance of magnetic levitation of protein crystals. We realized that we should explain more about the significance and engineering advantages of magnetic levitation of protein crystal growth. This would make the intent of this paper more easily understandable to readers. Therefore, we added the following sentence.

Pages 4-5, Lines 58-64.

"Growing protein crystals by levitation has an advantage other than the measurement of thermal properties. Generally, protein crystals are heavier than the solution, the crystals grow at the bottom of the container and often adhere to the bottom wall. The gas-liquid interface is a containerless condition [12-16], and it is expected that high-quality crystals will be produced. By growing them at the interface, we can easily pick them up without damaging them, which is convenient for structural analysis."

[3] Please provide the full expansion of the abbreviation PDB in the introduction.

[Res.]

Thank you for the advice. We revised the sentence as follows:

Page 4, Lines 46-47.

"Crystallographic details of thaumatin are made public in the Protein Data Bank (see the data of PDB entry 1RQW)".

[4] The introduction would benefit from a more comprehensive review of the relationship between understanding the thermal properties of proteins and the development of crystal growth technology.

[Res.]

Thank you for the advice. Proteins are well known to have a very high degree of supersaturation relative to their saturated solubility curves. Especially in that supersaturated state, temperature control has a significant impact on the crystal growth. Hence, we can deduce that thermal properties of protein crystal are essentially important knowledge for the progress of crystal growth technology.

In the revised version, we will cite a research paper by a group at Osaka University that developed an accurate temperature control machine, named TAON. This paper demonstrated that protein crystallization is clearly changed by the temperature. (The year after this paper was published, Maki transferred to this laboratory and made some contributions to the development of this research.)

Pages 4, Lines 55-58, the following sentence has been added:

"Such instruction will be useful for the development of crystal growth technology because we notice that temperature control has a significant impact on the crystallization [11] and thermal properties can make a contribution to the progress of temperature control."

11. Murai R., Nakata S., Kashii M., Adachi H., Niino A., Takano K., Matsumura H., Murakami S., Inoue

T., Mori Y., Sasaki T., Cooling-rate screening system for determining protein crystal growth conditions: *J. Cryst. Grow.* 2006, 292: 434–436, DOI: 10.1016/j.jcrysgro.2006.04.050

[5] In line 59, the authors should cite studies that confirm the presence of magnetic orientation of thaumatin.

[Res.]

In the previous version, we cited some research just before the sentence in page 5. Then, we omitted to present them there. In the new version, we refer to the papers of [19, 20] in Page 5, Line 69.

[6] The role of paramagnetic substances is not clear in the introduction.

[Res.]

The magneto-Archimedes effect is realized where the environment surrounding a diamagnetic substance is made paramagnetic and the repulsion of the magnetic forces acting on each other is used as magnetic levitation of diamagnetic substance (i.e., protein crystals). The amount of paramagnetic substance strengthens the repulsion, and the greater the amount (higher concentration), the smaller the magnetic force that can levitate the diamagnetic object. In actuality, protein crystals tend to aggregate with a large amount of paramagnetic substance. Thaumatin crystallizes when a smaller amount of paramagnetic substance ($GdCl_3$) is added than HEWL. Therefore, the only option was to strengthen the magnetic force. The mechanism of the levitation is explained in detail in the section of “Magneto-Archimedes levitation”; hence we cannot devote many pages to explaining it in the introduction.

In Page 5, Line 75, we added this short sentence:

“with a small amount of paramagnetic substance”

[7] To ensure consistency, I suggest that authors follow a uniform approach when referencing figures in the text.

[Res.]

Thank you for the comment. In the previous version, we were consistent in using “Figure” at the beginning of a sentence and “Fig. or Figs.” in the middle of the sentence. In the present version, we will standardize everything to “Figure”.

[8] Please explain in the paper how you calculated the magnetic field and the magnetic force in FIG1(d), and 1(e).

[Res.]

The distributions of ***B*** and ***F*** in Figure 1 and in Figure 2 were calculated with Maki’s original program code of FORTRAN, developed in 2003. This code can simulate the magnetic field distributions around

solenoidal circular coils. That is, this program approximates the actual shape of a superconducting magnet to that of a solenoidal electromagnet. Maki has utilized this program for many years and sometimes refined the code to progress his research on the magneto science.

To begin with, the magnet bore is divided into an equally spaced grid in a cylindrical coordinate system, and the center of each grid is defined as a scalar point. Next, a single circular coil is assumed to simulate a superconducting magnet coil. The one-turn circular coil is divided into equal infinitesimal line elements. The magnetic flux density acting between the line element and the scalar point is calculated using the Biot-Savart law. By calculating in the radial, circumferential, and axial directions, the magnetic flux density vector can be calculated. This process is repeated between each infinitesimal line element and its scalar point. This calculates the magnetic flux density vector $\boldsymbol{B}$ created by the one-turn circular coil at that scalar point. Since the calculation is expressed as a complex triple integral formula, the Simpson method is used for analytical approximation.

By assuming multiple circular coils with different diameters and positions, the magnetic field with multi-layer coils can be simulated. Its calculation process is similar to the case of a one-turn circular coil. By changing the scalar point, it is possible to calculate the magnetic field vectors even if the vessel is set up to an off-centered position from the central axis of the bore. Finaly, the magnetic force vector $\boldsymbol{F}$ is calculated using the magnetic flux density vector $\boldsymbol{B}$.

The actual shape and size of a commercial superconducting magnet are concealed by industrial secrets, and its detailed structure has not been made public. The calculation results by our program code are almost identical to the actual performance. The first research with this code is shown below. We will add this paper to the references. The following explanation has been added to the main text.

Pages 9-10, Lines 154-157.

"The distribution of the magnetic flux density vector $\boldsymbol{B}$ and the magnetic force vector $\boldsymbol{F}$ were obtained by the calculations using an original calculation code developed by the authors. In this program, the magnetic flux density is calculated using the Biot-Savart law [36]. Details are explained in previous studies [37, 38]."

36. Landau L. D., Lifshitz E. M., *The Classical Theory of Fields 4th Edition, Course of Theoretical Physics Vol. 2,* Pergamon Press, p. 103, ISBN 0-08-025072-6.

37. Maki S., Ataka M., Suppression and promotion of convection in water by use of radial components of the magnetization force, *J. Appl. Phys.* 2004, 96: 1696–1703, DOI: 10.1063/1.1763239

38. Maki S., Ataka M., Effects of non-axisymmetric magnetization force on natural convection of water at various off-centered positions in a superconducting magnet: Numerical computation studies, *Jpn. J. Appl. Phys.* 2005, 44: 1132–1138, DOI: 10.1143/jjap.44.1132

[9] Please provide appropriate citations to support the statement in line 153 "In most previous studies, magnetic orientation of thaumatin crystals was examined in a small capillary tube".

[Res.]

Thank you for the advice. We provided appropriate citations in the first sentence of the section

“*Equipment for the magnetic orientation*” in Page 10, Line 170.

Other revisions

[10] Six new papers have been cited. The citation numbers have been revised accordingly.

[11] The captions for Figures 1 and 2 have been updated to explain the derivation of the numerical results.

Response to the Editors

[12] We wish that our two types of short movies (“Short movie of Figure 4”, and “Short movie of Figure 5”) will be opened by the system of “Uploaded as supplementary information” of this journal. This comment was presented in Page 22 and 23 of the MS, as highlighted in green.

[13] Repositories

a) Can you make a de-identified, anonymized, or aggregated data set publicly available in a repository or, if that is not possible, in your manuscript’s Supporting Information files?

b) Are there any institutional representatives who did not collaborate in the study and are not listed as authors on the manuscript who would be able to hold the data and respond to external requests for data access? If so, please provide their contact information (i.e., email address). Please also provide details on how you will ensure persistent or long-term data storage and availability. For instance, data might be stored in two independent locations.

[Res.]

The short movies (“Short movie of Figure 4”, and “Short movie of Figure 5”) are opened in the website of the library of Okayama University of Science, as the repository.

https://ous.repo.nii.ac.jp/records/2000296

This library has institutional representatives who did not collaborate in the study and is not listed as authors on the manuscript who would be able to hold the data and respond to external requests for data access.

This website is also available in English, but when you access it, the Japanese version is displayed first. We have attached a PDF file showing how to switch to the English version. The file of which is “How to download the movies”.
